# Sequence Control from Mixtures: Switchable Polymerization Catalysis and Future Materials Applications

**DOI:** 10.1021/jacs.1c03250

**Published:** 2021-06-30

**Authors:** Arron C. Deacy, Georgina L. Gregory, Gregory S. Sulley, Thomas T. D. Chen, Charlotte K. Williams

**Affiliations:** Department of Chemistry, Chemistry Research Laboratory, 12 Mansfield Road, Oxford, OX1 3TA, U.K.

## Abstract

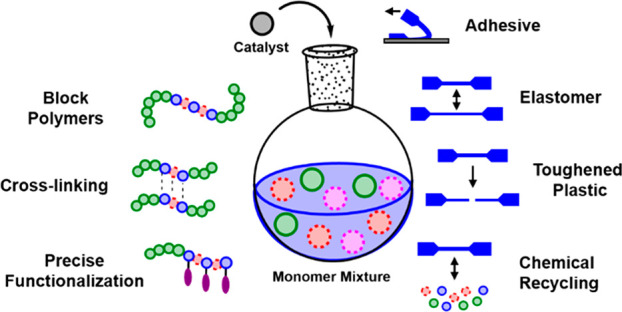

There is an ever-increasing demand for higher-performing polymeric materials counterbalanced by the need for sustainability throughout the life cycle. Copolymers comprising ester, carbonate, or ether linkages could fulfill some of this demand as their monomer–polymer chemistry is closer to equilibrium, facilitating (bio)degradation and recycling; many monomers are or could be sourced from renewables or waste. Here, an efficient and broadly applicable route to make such copolymers is discussed, a form of switchable polymerization catalysis which exploits a single catalyst, switched between different catalytic cycles, to prepare block sequence selective copolymers from monomer mixtures. This perspective presents the principles of this catalysis, catalyst design criteria, the selectivity and structural copolymer characterization tools, and the properties of the resulting copolymers. Uses as thermoplastic elastomers, toughened plastics, adhesives, and self-assembled nanostructures, and for programmed degradation, among others, are discussed. The state-of-the-art research into both catalysis and products, as well as future challenges and directions, are presented.

Nature applies its synthetic (bio)chemistry to reagent mixtures, exquisitely controlling life’s complex milieu to make sophisticated, reconfigurable, and responsive products. A triumph of twentieth-century synthetic chemistry has been the multitude of highly selective reagent transformations, many operating at large-scale, to form specific products. One future vision is to develop synthetic chemistry using raw material mixtures, perhaps even with variable or changeable composition or purity, through reconfigurable transformations to yield different and responsive products from a single process reactor or manufacturing plant. Such chemistry is conceptually appealing, but of course very challenging; it could be both sustainable and economically attractive by maximizing raw material usage, reducing purifications/separations, reconfiguring existing manufacturing plants, developing products responsive to society’s needs, and improving energy efficiency.^1,2^

Block polymers provide a platform to produce a diverse variety of materials, with applications as advanced materials, in drug delivery and in nanolithography, among others. The joining of homopolymers into block structures allows for the fine-tuning of physicochemical properties. Microphase separation of the blocks enables materials that combine the properties of the respective homopolymers to furnish a new set of features. For example, poly(styrene-*b*-butadiene) is a thermoplastic elastomer which beneficially combines the elasticity of poly(butadiene) with the high stiffness of poly(styrene).

This perspective focuses on a nascent catalytic process that can synthesize sequence-controlled block polymers from mixtures of monomers. Switchable polymerization catalysis applies mixtures of monomers and uses a single catalyst that accesses different catalytic cycles to produce block sequence-selective copolymers.^3−6^ In this catalysis, “switches” refer to the ability to direct the catalyst *between different* polymerizations while selecting for particular monomers (Figure 2a). It is a specific form of autotandem catalysis and allows for coupling of heterocycle/heteroallene ring opening copolymerizations (ROCOP) with heterocycle ring opening polymerizations (ROP). Regarding the terminology ‘switchable polymerization catalysis’, there are other terms used in this field that refer to the same process, including “mechanism switch”, “self-switch”, or “smart switch”. In spite of the different names, all these catalyses follow the same general principles and rules. Further, these switchable polymerizations are different to orthogonal tandem catalyses, where multiple catalysts operate consecutively or in concert, each optimized for a specific process. They also differ from assisted-tandem catalyses, coined “on/off *exogeneous* switches”, where external triggers result in a change to the catalyst active species. Such triggers include chemical,^7−12^ mechanical,^13^ thermal,^14^ electrical,^15−17^ or photochemical^19−23^ and are thoroughly reviewed elsewhere.^24−26^ Such assisted-tandem catalyses can also be used to prepare block polymers; for example, Diaconescu and co-workers exploited an efficient ferrocene redox couple to control ring-opening polymerizations between ε-caprolactone or lactide enchainment (Figure 2b).^9,11^

The science of switchable polymerization catalyses is still at the outset, and much remains to be discovered, properly understood, and fully optimized. This review recounts its discovery, mechanisms, and catalytic control and highlights opportunities, as well as future challenges, for the resulting processes and polymers (Figure 1). Here, we focus exclusively on the coupling of heterocycle/heteroallene ROCOP and heterocycle ROP catalytic cycles resulting in the self-switching of monomer mixtures, i.e. where the addition or removal of monomers dictates the catalysis and polymerization pathway. The mechanistic switch can be designed to occur passively based on the principles of living polymerization, i.e., from monomer consumption and by using predetermined monomer ratios. Alternatively, the switches can be initiated actively by adding or removing monomers such as carbon dioxide or anhydrides (Figure 2a). Its use builds up complex macromolecular structures without any need for other changes to conditions or external triggers, e.g. electrochemistry/light. It obviates the need for protection/deprotection steps or intermediate purifications which are commonplace when different polymerization mechanisms are used to build up a block polymer.

**Figure 1:**
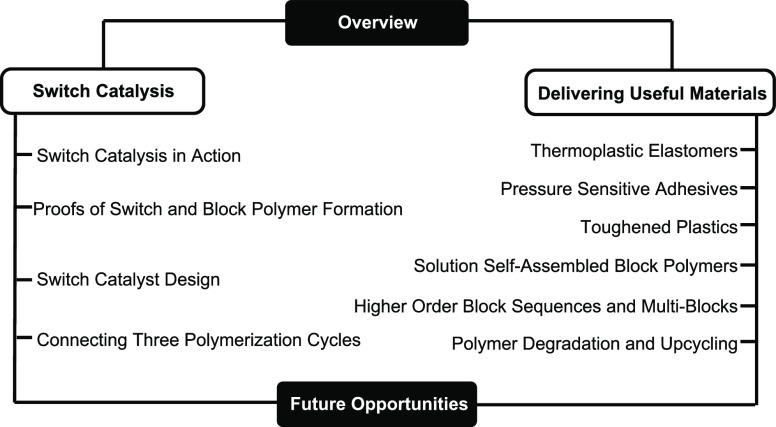
Contents of the perspective.

**Figure 2:**
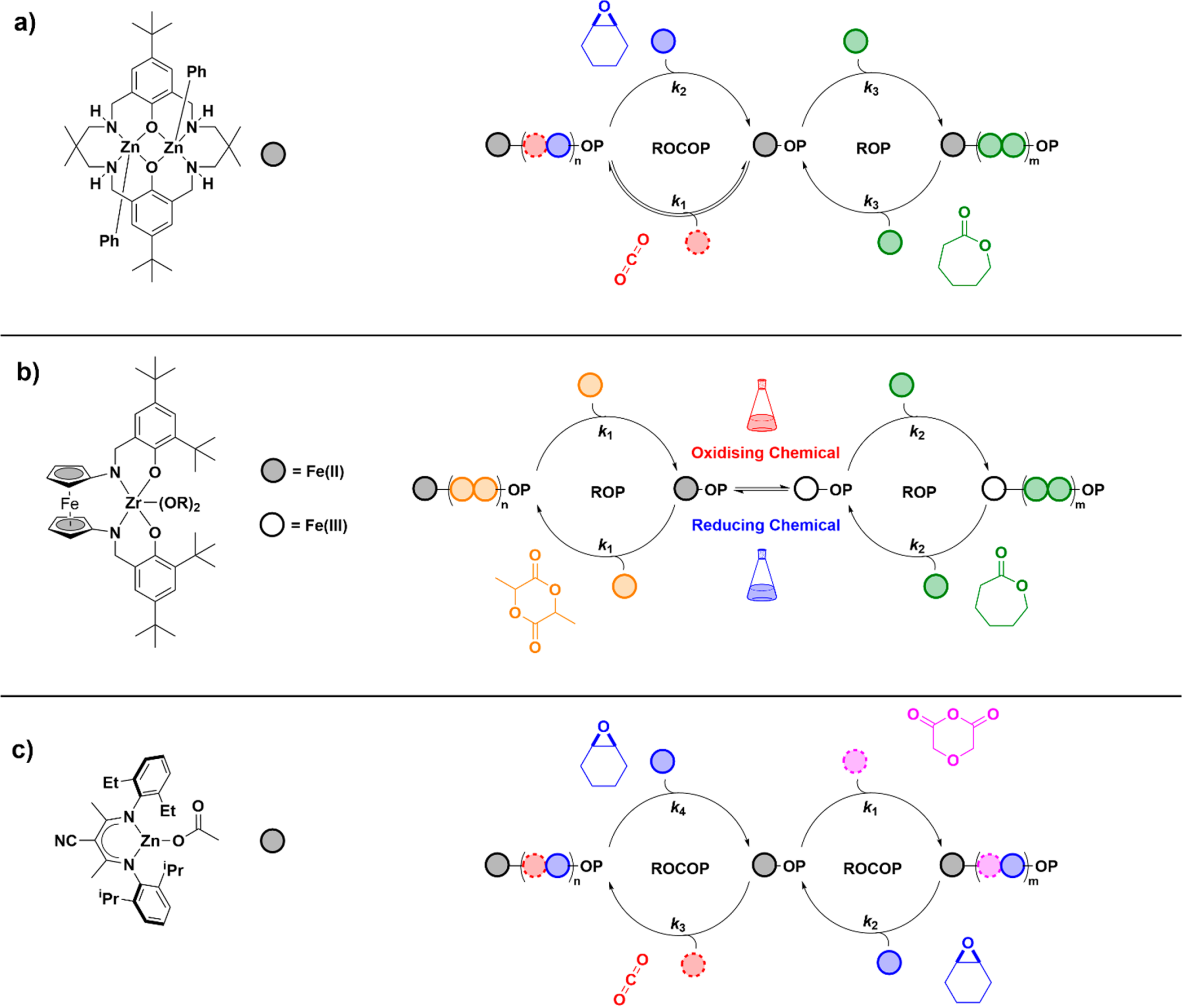
(a) Mechanistic switches achieved through presence/absence of monomers. (b) Redox Switch in cyclic ester ROP through chemical oxidation/reduction of the catalyst. (c) Terpolymerization of anhydride, epoxide, and carbon dioxide using a zinc-β-diimine catalyst.^3,9,11,18^

These switches, driven both kinetically and thermodynamically, often allow for complete and instantaneous change from one block “sequence” to another. This feature alleviates difficulties manipulating different monomer reactivity ratios, carefully timed anaerobic reagent “additions” or formation of tapered structures. The catalysis also delivers diverse copolymer sequences and is compatible with different polymerization mechanisms as well as backbone and side-chain polymer chemistries.

Prior to describing these catalyses, it is appropriate to consider the features of the under-pinning controlled heterocycle polymerizations, i.e. (1) cyclic ester/ether/carbonate ring-opening polymerization (ROP) and (2) heterocycle/heteroallene ring-opening copolymerization (ROCOP). Heterocycle ROP is driven by relief of ring strain and, for cyclic esters/carbonates, functions most effectively using 4-, 6-, and 7-heterocycles, while for cyclic ethers 3–5-membered rings are polymerizable. It is used commercially to produce aliphatic polymers, including biobased polyesters, and this catalysis is generally well understood and optimized.^27−30^ Its detractions are low polymerization equilibria for substituted or functionalized cyclic monomers, functional group intolerance, and limited commercial/large-scale production of many monomers.^31−33^ In contrast, epoxide/heteroallene ROCOP operates effectively using functionalized, semiaromatic, and rigid monomers. Many epoxides and cyclic anhydrides are produced and used at scale, within a cost-range acceptable to the production of polymers.^34^ This copolymerization catalysis is less developed and, except for carbon dioxide/epoxide ROCOP, still suffers from low rates, selectivity, and molar mass, and requires high catalyst loadings.

The first catalyst able to conjoin both epoxide/cyclic anhydride ROCOP and lactone ROP pathways was reported by the pioneering team of Inoue and co-workers in 1985.^35−37^ In their ground-breaking report, an Al-porphyrin catalyst system produced block oligoesters by sequential phthalic anhydride/propylene oxide ROCOP, followed by β-butyrolactone ROP.^37^ This chemistry was not described as a polymerization catalysis “switch” and was not revisited for several decades. In the intervening period, significant advances occurred in the activity and selectivity of epoxide/heteroallene ROCOP catalysts.^38−45^ Another ground-breaking report, from Coates and team in 2008, demonstrated that epoxide, carbon dioxide, and anhydride ROCOP, i.e. terpolymerization, occurred with unparalleled selectivity (Figure 2c).^18^ The epoxide/anhydride ROCOP occurred selectively to make polyester before any formation of polycarbonate (epoxide/carbon dioxide) block, even though the reactivity order was the converse; this unexpected finding was rationalized by kinetic control. Subsequently, there were a few reports of polymerizations using mixtures of lactones, epoxides, and heteroallenes, some using heterogeneous catalysts, but the catalysis was poorly controlled and lacked the key evidence for a mechanistic switch and/or block sequence selectivity.^46−50^ In 2014, we investigated homogeneous dizinc catalysts for both epoxide/carbon dioxide ROCOP and lactone ROP and discovered using mixtures of all monomers an unexpected orthogonal reactivity dependent upon the zinc-polymer end group chemistry.^3^ Zinc alkoxides reacted with carbon dioxide or lactones, but not with epoxides, and zinc carbonates reacted with epoxides, but not with lactones. The selectivity appeared likely to be thermodynamic in nature, since even under high temperatures or over extended periods insertions did not occur. However, by controlling the monomers present, the Zn-chain end group chemistry could be rapidly and reversibly “switched” into or out of specific polymerization pathways, thereby controlling the copolymer composition.

It is also appropriate to comment more generally on the copolymers’ molar masses and resulting application potential. Some catalysts deliver low molar mass hydroxyl telechelic oligomers by exploiting rapid and reversible chain transfer reactions, for example, with alcohols. Such hydroxyl functional oligomers are useful as low viscosity prepolymers for resins, in polyurethane manufacture and coatings, adhesives, and sealant applications.^51−58^ Switch catalysis may also deliver high molar mass polymers, and in some cases, block phase separation would be expected to follow the usual self-assembly theories.^59−62^ Thus, applications spanning elastomers, plastomers, adhesives, and self-assembled colloids are, or could be, feasible.

An attractive feature of these switchable catalyses is that the heteroatom rich polymers produced may show improved sustainability compared with conventional hydrocarbon polymers. Many switchable catalyses apply scalable, commercial, and, where feasible, bio- or waste-derived monomers.^63,64^ For polymer chemists, the allure of “new monomers” is strong, but we believe more polymer science should be uncovered using the "existing commercial monomer pool" and devising new “construction” chemistries to control sequence, stereochemistry, and architecture. Advances in monomer manufacturing are delivering biobased routes and integration of coproducts or wastes, reducing feedstock reliance on virgin petrochemicals.^34^ Polyesters, -carbonates and -ethers have monomer–polymer chemistries that are closer to equilibrium than today’s hydrocarbon polymers.^2^ Their chemistry, therefore, shows substantially lower energy requirements for chemical recycling, i.e. depolymerization to monomers. These linkage chemistries are in many cases susceptible to hydrolyses or biodegradation reactions, even if the rates of these reactions will be environment-dependent; this feature could be important to reduce pollution should waste systems fail or materials escape into the environment.^2,65^ Polymer sustainability must be life cycle assessed on a case by case basis, but these catalyses and chemistries could help deliver net-zero, circular economy and sustainability goals.

## Switch Catalysis in Action

**Figure 3:**
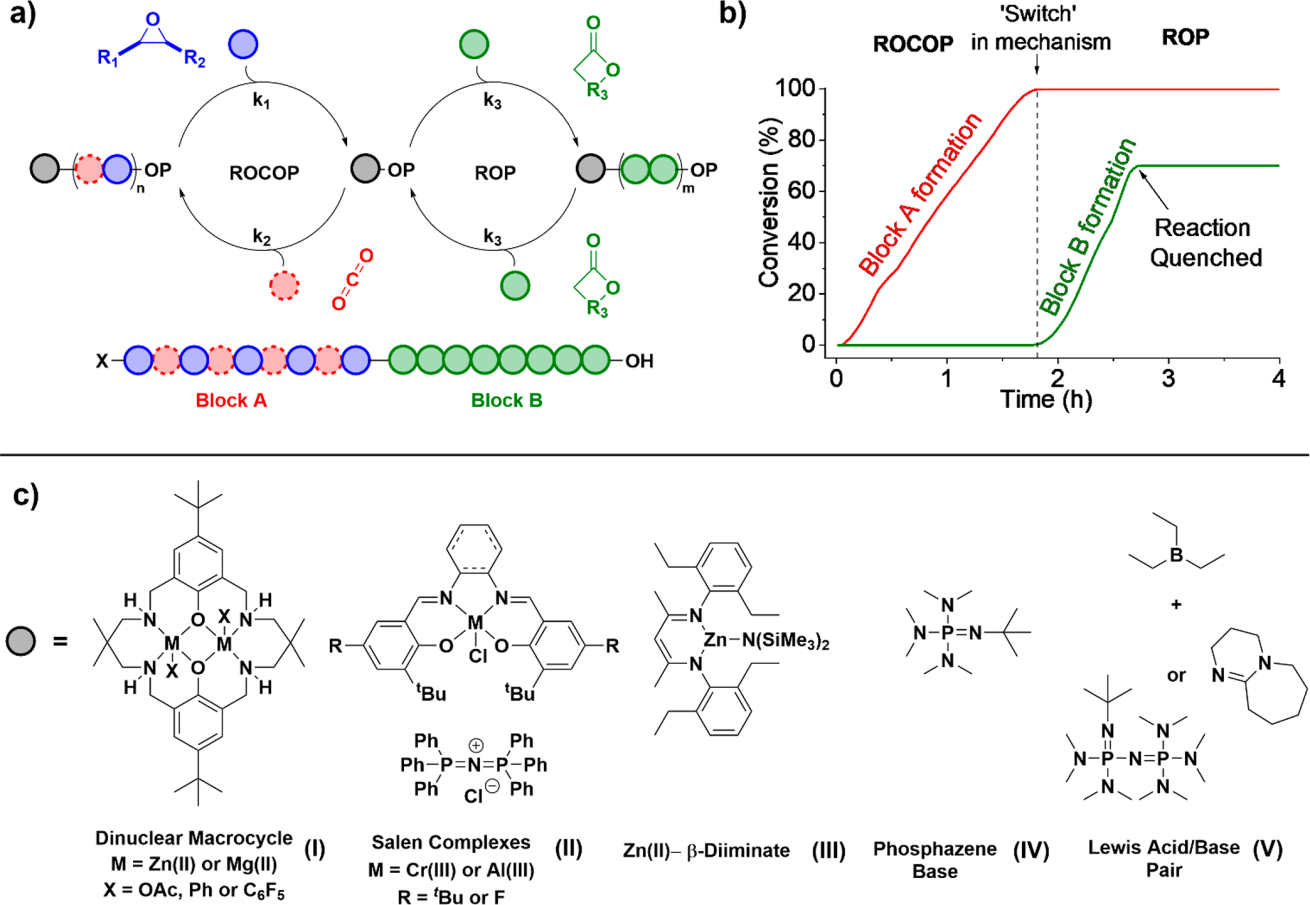
(a) Illustration of representative heterocycle ROCOP and ROP pathways. (b) Reaction conversion versus time plot showing the growth of each block. (c) Examples of switch catalysts.^3,4,6,56,66−78,80−84^

In 2014, a dizinc catalyst (**I**) was discovered to be able to link CO_2_/cyclohexene oxide (CHO) ROCOP and ε-caprolactone ROP forming block polymers (Figure 3).^3,66^ Although the ROCOP rate was an order of magnitude slower than that of ROP (∼7 h^–1^ vs ∼100 h^–1^), it proceeded first due to rapid carbon dioxide insertion chemistry (*k*_2_ is fast; ROCOP is zero order in carbon dioxide). The metal–carbonate intermediate formed may ring open an epoxide monomer (*k*_1_ slow) but not the surrounding lactone monomers. Thus, when CO_2_ was present complete selectivity for poly(cyclohexene carbonate) (PCHC) was afforded. After CO_2_ removal, a metal-alkoxide species was generated, which could ring open the lactone (*k*_3_) to form a metal-alkoxide that subsequently formed poly(caprolactone). The catalyst showed high ROCOP selectivity, avoiding cyclic carbonate or polyether linkage formation. Moreover, limited transesterification of the blocks occurred.

Following this discovery, the same switchable catalysis concept was demonstrated using mixtures of anhydrides/epoxides/lactones. All the reactions followed the same reactivity rules and allowed for selective formation of specific block polyesters (e.g., PDL-*b*-PE-*b*-PDL, where PE = poly(phthalic anhydride-*alt*-cyclohexene oxide), PDL = poly(ε-decalactone).^67^ Later, the same switchable catalysis rules were discovered to be generally applicable to many different catalyst systems, including dinuclear metal catalysts (**I**),^3,66−73^ metal salen catalyst/cocatalyst systems (**II**),^56,74−79^ Zn(II)-β-diiminates (**III**),^4,80^ and organocatalysts (**IV** and **V**).^6,81−84^ It is now possible to select a catalyst according to its activity, monomer scope, block selectivity, and loading/polymer molar mass. For instance, the dinuclear catalysts, **I**, are efficient when carbon dioxide incorporation is desired as reactions succeed at low pressure (1 bar), overcoming the need for specialized equipment. Organometallic dinuclear catalysts improve polymerization control making block polymers with molar masses up to 100 kg mol^–1^ and showing narrow dispersity (*Đ* < 1.1). One drawback of many of these catalysts is the lack of/lower reactivity using alkylene oxides, e.g. propylene oxide (PO).

Metal salen catalyst systems, **II**, have a broad monomer scope for epoxide/anhydride ROCOP and heterocycle ROP, and some catalysts are commercial products. Nonetheless, these catalysts remain under-explored for switchable catalyses involving carbon dioxide incorporation, perhaps because higher pressure equipment and analytical methods are likely required. Their major drawback is the requirement for a cocatalyst which complicates end-group fidelity and may prevent selective switch catalysis from occurring. The cocatalyst is also expensive, toxic, and may be corrosive to steel. β-Diiminate Zn(II) catalysts, **III**, require high-pressure CO_2_ (40 bar) for switch catalysis but form block polymers with high molar masses (>140 kg mol^–1^) and moderate/good control (*Đ* 1.2–1.8). This catalyst class also includes rare limonene oxide/CO_2_ ROCOP catalysts, relevant because limonene may be extracted from waste citrus fruit peel. However, such catalysts only polymerize *trans*-limonene oxide from a commercial *cis*/*trans* mixture (40:60); hence significant unreacted limonene oxide remains.^85,86^ Other detractions for catalysts **III** include a high ligand susceptibility to alcoholysis or hydrolysis reactions necessitating rigorously anhydrous reagents, lack of clarity regarding end-group fidelity, and difficulties accessing low molar mass polyols (where excess diol is necessary). Phosphazene bases or Lewis acid/base pairs (organocatalysts), such as **IV** and **V**, are straightforward to apply and function with a range of monomers for epoxide/anhydride ROCOP and heterocycle ROP. These catalysts remain largely unexplored for switchable polymerizations using carbon dioxide. So far, these catalyst systems are applied at high loadings and resulting polymer molar mass values are limited to <30 kg mol^–1^ (*Đ* < 1.2). It is important to note that some of these organocatalysts are very expensive, toxic, and appear complex to use at scale. On the other hand, recent reports of related organo-catalyst heterogenization and recycling for epoxide/carbon dioxide ROCOP could be an exciting development for switchable catalysis.^87,88^

## Proofs of Switch and Block Polymer Formation

Key to the successful development of these switchable catalyses is the experimental proofs of both monomer selectivity and block polymer formation. Switch catalysis selectivity is best established using *in situ* spectroscopic reaction monitoring.^79^ FTIR spectroscopy is particularly useful since it distinguishes polycarbonates (ca. 1250 cm^–1^), polyesters (ca. 1060 cm^–1^), and cyclic carbonates (ca. 1820 cm^–1^) and its high sensitivity improves accuracy. It also prevents any changes to the reaction conditions (e.g., reduced CO_2_ pressure or stirring differences), which are unavoidable when using *in situ* NMR spectroscopy to monitor reactions.^69^ As illustrated in Figure 3, *in situ* spectroscopy is invaluable to establish selective catalyses and to fully understand the “stages” of different monomer enchainment. Occasionally catalysts fail to switch cleanly and concurrently access two different catalytic cycles—this results in the formation of tapered or even random copolymers.^4,56^ While it might be expected that such selectivity breakdown would be common, in fact, it occurs surprisingly rarely.

*In situ* spectroscopy alone cannot establish pure block polymer formation. Block attachment must be ascertained by different methods as mixtures of unattached homopolymers formed sequentially would also show perfect monomer selectivity if only analyzed by *in situ* spectroscopy. No single technique is sufficient to prove block attachments, but rather several methods should be used together including: (1) End group analysis by ^31^P NMR spectroscopy after addition of a phosphorus capping reagent; (2) Evolution of molar mass and distribution(s), using size exclusion chromatography (SEC); (3) Identification of block junction resonances, using NMR spectroscopy (^1^H, ^13^C NMR, COSY); (4) Testing of a single diffusion coefficient for all NMR resonances and comparison with the diffusion coefficients observed for mixtures of homopolymers (DOSY); (5) Characterization of block polymer thermal properties by thermal gravimetric analysis (TGA) and differential scanning calorimetry (DSC); and (6) Attempts to change polymer composition using physical fractionation methods (solvent precipitation, dialysis, or column chromatography).

## Switch Catalyst Design

Catalyst selection fundamentally controls the switchable polymerization activity, selectivity and substrate scope. To drive progress, criteria for “excellent” switch catalysts are outlined:

1. *High performance*. The best catalysts are fast, selective, and well-controlled for all heterocycle ROP and ROCOP stages. Successful catalysts show minimal/no side-reactions, including preventing transesterification or other block scrambling reactions.
2. *Simplicity*. Catalysts should be straightforward to synthesize and avoid the use of cocatalysts (e.g., bis(triphenylphosphine)iminium chloride, PPNCl). Many heterocycle ROCOP catalysts rely on these cocatalysts, but, unfortunately, they complicate the polymerization kinetics, limit useable catalyst loadings, and disturb end-group fidelity.
3. *Monomer scope*. Catalysts should enchain many different monomers and yield polymers with easily controllable chain rigidity, crystallinity, and side-chain functionality. Use of biobased or waste recycled monomers, including those with low purity grade, is also a priority for catalyst development.
4. *Sequence selectivity*. Highly selective and controlled catalysts access multiblock structures with different architectures. Catalysts must be stable, including in the presence of a large excess of chain transfer agent (CTA) to control chain compositions and molar masses; e.g. monoalcohol leads to AB, and diol, to ABA sequences, and triol/tetraol, to stars with sequence control. Catalysts must maintain activity and selectivity through multiple monomer additions to build up the most sophisticated sequences and patterns.
5. *End-Group fidelity*. Both chain ends must be fully controlled to cleanly access specific chain sequences. Only the best catalysts are fully end-group selective but are essential to access pure block polymers.
6. *Processability* . Catalysts should maintain activity, tolerate lower purity monomers, and be easily removed from the product and/or recycled. They should show low toxicity and be sourced from abundant metals/resources.

**Figure 4:**
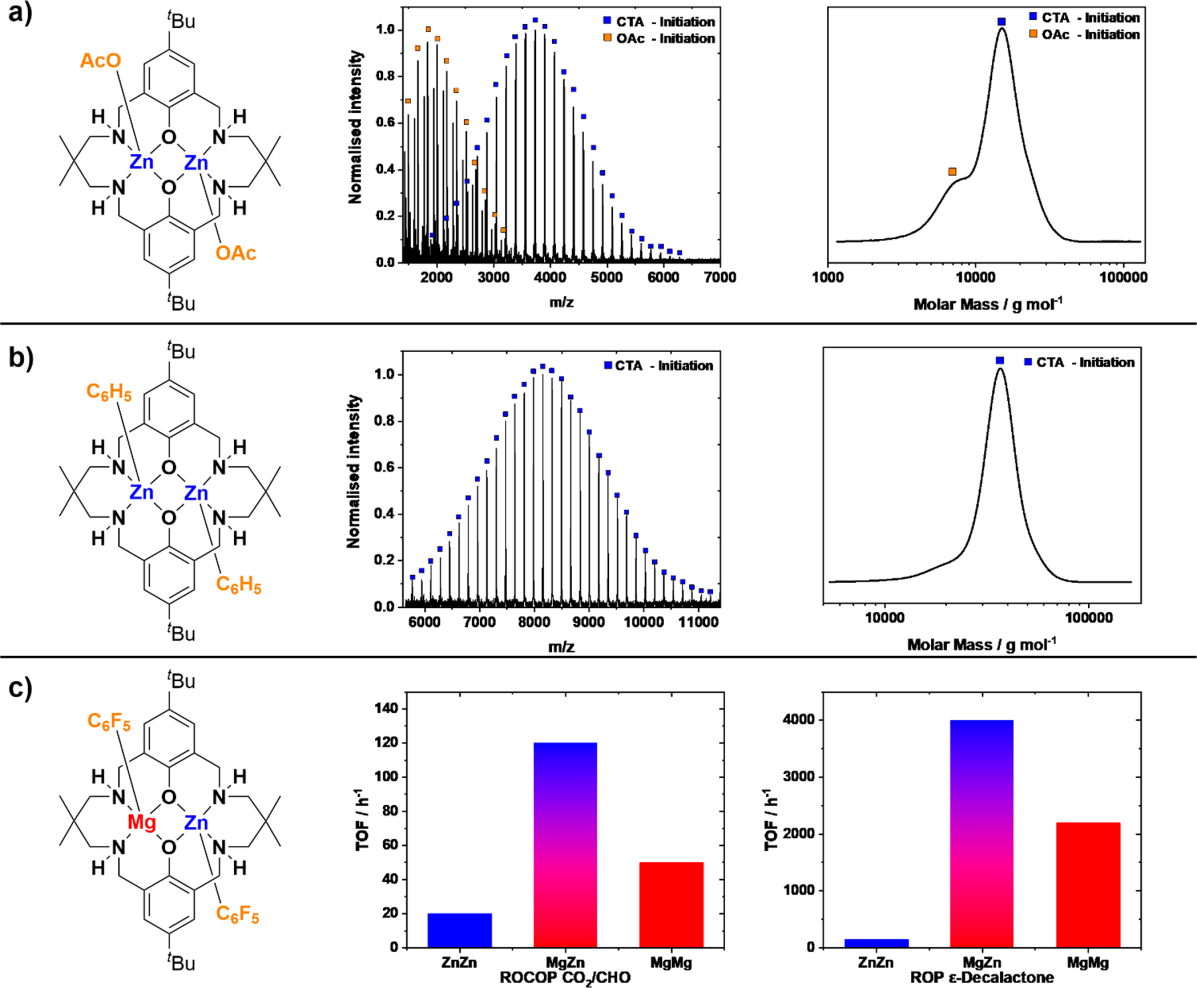
All data displayed above is for the reaction of CO_2_/cyclohexene oxide (80 °C, 0.1 mol %, 4 equiv of CTA (1,2-cyclohexane diol), 1 bar CO_2_) unless stated otherwise. (a) Reaction outputs using a dizinc di(acetate) catalyst that results in a mixture of polymer end-groups and bimodal molar mass distributions when applied with 1,2-cyclohexane diol). (b) Reaction outputs using adizinc di(phenyl) catalyst, applied with 1,2-cyclohexane diol, that controls end-groups and yields monomodal molar mass distributions. (c) Reaction outputs using a heterodinuclear MgZn di(pentafluorophenyl) catalyst showing high end-group fidelity, monomodal molar mass distributions, and faster rates for both ROCOP (CO_2_/CHO) and ROP (ε-decalactone) than the dizinc or dimagnesium analogues (under identical, unoptimized conditions).^67,71,89^ Image adopted with permission from ref (71). Copyright 2020 American Chemical Society.

To exemplify catalyst development for these switchable polymerizations, the first dizinc catalysts were improved by changing both the metals and the initiating groups or coligands (Figure 4).^89^ The first di-Zn(II) catalysts showed good selectivity but relatively low rates and high catalyst loadings, limiting the polymer molar mass and causing bimodal molar mass distributions (Figure 4a).^3^ To tackle these shortcomings, an organometallic dizinc catalyst was designed featuring phenyl initiators. This catalyst was applied with alcohol as a chain transfer agent to generate the true initiator *in situ* (a zinc alkoxide species). This catalyst shows excellent end-group selectivity and yields monomodal molar mass distributions (Figure 4b).^90^

Nonetheless, its rates were still low (TOF_ROCOP_ = 20 h^–1^, 0.1 mol %, 80 °C, 1 bar CO_2_). Next, a heterodinuclear Mg(II)/Zn(II) catalyst, also featuring an organometallic ligand and applied with chain transfer agent, showed enhanced rates (TOF_ROP_ = 4000 h^–1^; TOF_(ROCOP)_ = 120 h^–1^, 0.1 mol %, 80 °C, 1 bar CO_2_).^71,91^ The Mg(II)/Zn(II) catalyst shows synergy between the two metals and because it features organometallic initiators shows the highest end-group fidelity. It was used to prepare high molar mass ABA block polymers selectively; these showed phase-separated microstructures (A = poly(cyclohexene carbonate); B = poly(ε-decalactone) (*vide infra*)).^71^ Further improvements to activity, loading, selectivity, and tolerance are anticipated through modifications to the catalyst structure. The stratagem of using organometallic ligands, such as alkyl or aryl groups, should also be more broadly explored, as it confers the best end-group control and maximizes molar mass values.

## Connecting Three Polymerization Cycles

**Figure 5:**
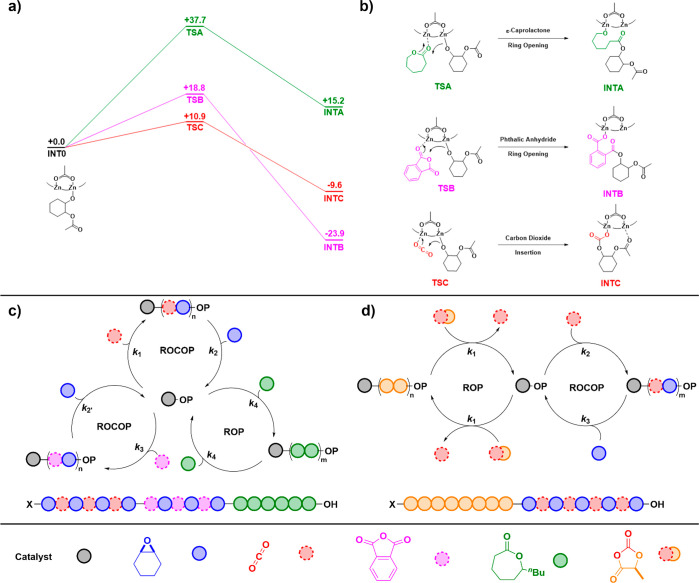
(a) DFT (Δ*G* kcal mol^–1^) energy profiles for different monomer insertions into a zinc-alkoxide intermediate: CO_2_ (red), phthalic anhydride (pink) and caprolactone (green). (b) Illustrations of transition states and postinsertion intermediates. (c) Switch catalysis connecting three polymerization cycles: anhydride/epoxide ROCOP, lactone ROP, and CO_2_/epoxide ROCOP. (d) Switch catalysis connecting two polymerization cycles: *O*-Carboxy anhydride (OCA) ROP followed by ROCOP of CO_2_ (released from OCA ROP) and CHO.^68−70^

Most reports of switch catalysis join two catalytic cycles and produce AB or ABA structures, but with the right catalysts, three different cycles can be joined to make higher block sequences. A combined kinetics and DFT study of dizinc catalyst, **I**, revealed a relative monomer insertion order of anhydride > carbon dioxide > lactone into the dizinc alkoxide intermediate (Figure 5).^68^ Although the barrier to anhydride insertion (18.8 kcal mol^–1^) is higher than that of CO_2_ (10.9 kcal mol^–1^), the anhydride is consumed before CO_2_. This observation is rationalized by the different stabilities of the insertion products (carboxylate = −23.9 kcal mol^–1^, carbonate = −9.6 kcal mol^–1^) (Figure 5a, b). The barrier to carbon dioxide extrusion from the carbonate intermediate is similar to the epoxide ring-opening barrier (decarboxylation: = 21 kcal mol^–1^ vs epoxide ring-opening = 22.9 kcal mol^–1^). In contrast, anhydride elimination from the metal-carboxylate is not feasible (anhydride elimination = 32.5 kcal mol^–1^). Thus, selectivity is determined by the potential for reversible CO_2_ insertion and the overall linkage stabilities. Experiments targeting ABC block polymers from mixtures of 4 monomers showed that, after CO_2_ removal, backbiting of the poly(cyclohexene carbonate) block occurred, extruding cyclohexene carbonate, rather than the expected enchainment of the polylactone.^69^ This side-reaction was resolved by changing the order of monomer addition to build up an ABCBA pentablock polymer by epoxide/anhydride ROCOP, followed by lactone ROP and completed with CO_2_/epoxide ROCOP (Figure 5c). Another example of ABCBA pentablock formation involved propylene oxide (PO)/anhydride ROCOP, PO ROP, and ε-decalactone ROP; in this case, the Cr(III) catalyst system can access epoxide ROP cycles, a process inaccessible to the class **I** catalysts, demonstrating the importance of catalyst selection.^56^*O*-Carboxyanhydride (OCA) ROP provides an alternative route to poly(lactic acid) (PLA), the quintessential biodegradable polyester, and with each monomer insertion, an equivalent of carbon dioxide is released.^92^ A one-pot switchable catalysis recycled the released carbon dioxide to form poly(l-lactide-*b*-cyclohexene carbonate) (Figure 5d).^70^ In this case, the reaction relies upon the dizinc catalyst **I**, which can enchain at low CO_2_ pressures (<1 bar). Overall, ∼91% carbon recycling into polymer was achieved.

## Delivering Useful Materials

Switch catalysis delivers multiblock polymers, many of which would be difficult, or even impossible, to make by other means. It diversifies the palette of degradable and bioderived polymers without requiring multiple new monomers. Any new material must deliver equivalent or better properties than incumbent petrochemicals. So far, proof of concept applications such as thermoplastic elastomers (TPEs), pressure-sensitive adhesives (PSAs), and toughened plastics highlight the potential for these new products made by switch catalysis.

Generally, block polymers show mechanical, thermal, and rheological properties dependent on chemical structure, and in some cases, properties are better when phase separated microstructures are accessed.^61,93,94^ The phase separated morphology depends upon block incompatibility (expressed as the Flory–Huggins interaction parameter, χ), the overall degree of polymerization (*N*), block composition (expressed as a volume fraction, *f*), and architecture (linear, star, etc.). Morphologies are also influenced by block dispersity and polymer processing history.^95,96^

**Figure 6:**
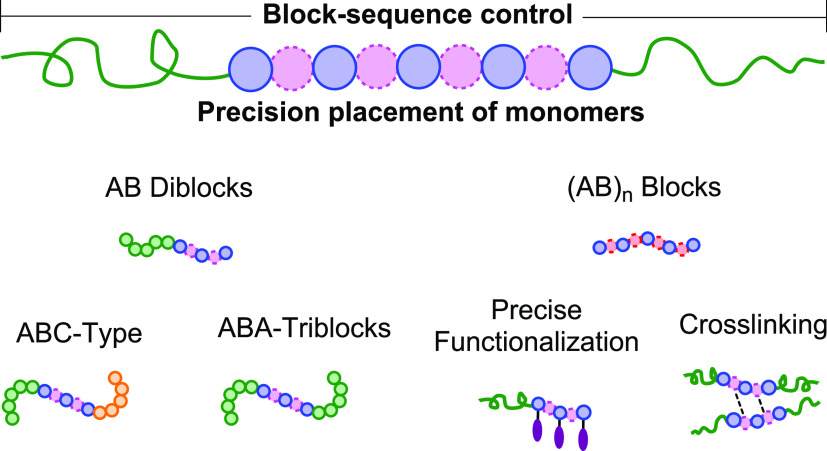
Overview of some of the block and precision structures accessible by switchable polymerization catalyses.

At the polymer chain level, the comonomer sequence also affects both properties and overall degradation rates.^97−101^ Heterocycle ROCOP is notable for the formation of highly alternating monomer sequences (Figure 6).^44,45,102^ Such precision carbonate or ester linkage placements, even in short segments, could have marked physicochemical implications. Alternating functionalized monomer sequences, albeit attached to different polymer backbones, have already shown strong donor–acceptor photophysical interactions or delivered specific binding sites for biochemicals relevant to targeted therapeutic delivery.^103−105^ There may also be long-term options to exploit precision monomer sequences for chemical data storage, to direct chain folding to mimic biopolymer structures or to furnish sites for dynamic cross-linking and vitrimers.^106−108^

## Thermoplastic Elastomers

Thermoplastic elastomers (TPEs) typically comprise ABA block polymers, where A = rigid, glassy/semicrystalline block (*T*_g_ or *T*_m_ > rt) and B = elastomeric soft block (*T*_g_ < rt). Phase separation into hard and soft domains is crucial to deliver the best mechanical properties; spherical or hexagonally close-packed cylinder morphologies generally correlate with elastomeric behavior (linear stress–strain plots).^59,61,109^ Thermally reversible physical cross-linking, resulting from block phase separation, means these elastomers are recyclable and reprocessable with retained thermoplastic behavior, which is in contrast to chemically cross-linked thermosets. Common commercial thermoplastic elastomers include petroleum-derived styrenics, such as poly(styrene-*b*-butadiene or isoprene-*b*-styrene) (SBS and SIS). Although already successful in many applications, some property limitations remain. For instance, their upper operating temperature must be below 90 °C and they are not degradable. Hillmyer and co-workers pioneered alternative all polyester-based TPEs, exploiting semicrystalline PLA hard domains coupled with flexible biobased polymers, from castor oil, peppermint, or sugars.^31,62,110^ Inspired by this chemistry and the elastomeric properties achieved, we have used switchable catalysis to deliver block polyesters from PA/CHO/DL mixtures.

**Figure 7:**
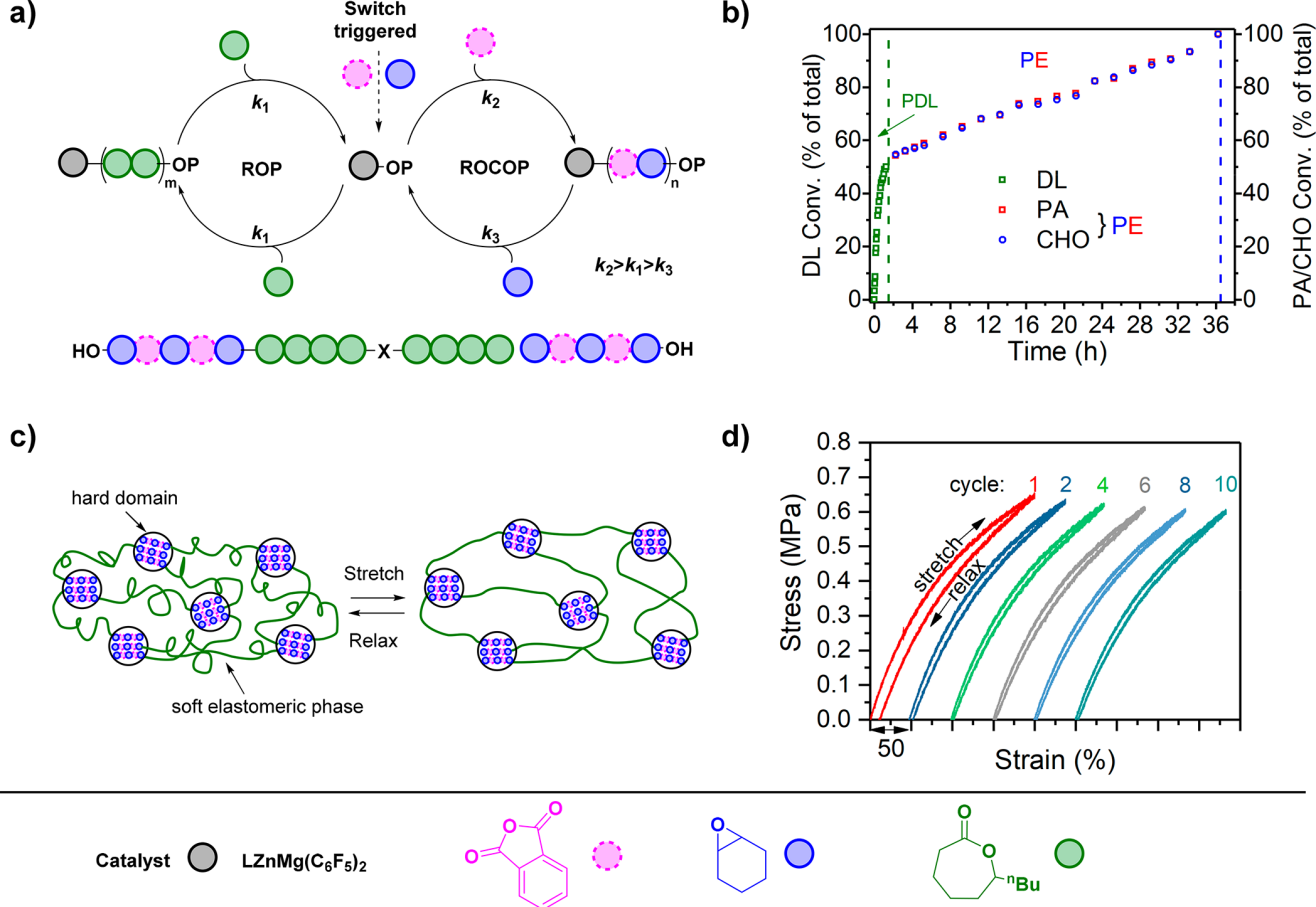
Thermoplastic elastomers by switch catalysis, exploiting the rigid structures of polyesters prepared by ROCOP as hard domains for physical cross-linking and amorphous elastomeric polyesters prepared by lactone ROP. (a) Switch catalysis illustration of ε-decalactone ROP (green) and ROCOP of phthalic anhydride (red)/cyclohexene oxide (blue). (b) Conversion–time plot of switch reaction monitored at regular intervals by ^1^H NMR spectroscopy. (c) Schematic illustrating the stretching and relaxing of triblock copolymer. (d) Stress–strain plot of elastomer undergoing stretching and relaxing with minimal hysteresis loss over 10 cycles.^72^ Image adopted from ref (72) with permission from The Royal Society of Chemistry.

In terms of monomer sourcing, DL comes from castor oil, and routes to PA from corn stover and CHO from 1,4-cyclohexadiene, a waste product of plant oil self-metathesis, have been reported.^111−113^ Using mixtures of these three monomers results in PA/CHO ROCOP, followed by DL ROP and yields unconventional soft-*b*-hard-*b*-soft (BAB) architectures.^114^ The relatively high block miscibility (low χ) requires a high *N* for phase separation. In the first experiments, hydroxyl-telechelic BAB chains were extended, by coupling with diisocyanates, to deliver elastomeric, plastic, and even shape memory properties. Subsequently, exploiting an improved catalyst and sequential monomer addition strategy delivered high yields of ABA triblocks.^71^ These materials showed *M*_*n*_ ≈ 100 kg/mol and provided sufficiently high *N* to show phase separated microstructures and eliminate any need for chain extension (Figure 7a, b). The resulting all-polyester TPEs showed high elongation at break (up to 1900%), moderate Young’s Moduli (1.5–5.0 MPa), and tensile strength (2.0–6.5 MPa); performances were competitive with those of commercial SIS elastomers. Repeated extension/relaxation experiments (200% extension) demonstrated little hysteresis loss and high elastic recovery (>95%) (Figure 7c, d). These polymers showed excellent stability but were also readily degradable under moderately acidic conditions (pH 4). Another advantage lies in the use of rigid polyester PA/CHO ROCOP blocks which increase the upper service temperature (140–160 °C) exceeding those accessible using PLA (55–60 °C) or PS (90 °C) hard domains.

Recent reports of block polycarbonate TPEs, although not prepared using switch catalysis, indicate the potential for other CO_2_/epoxide ROCOP hard or soft blocks.^115,116^ Feng and co-workers conducted 1-octene oxide/CO_2_ ROCOP, using organocatalysts, to afford a soft PC block (*T*_g_ −24 °C) and poly(cyclohexene carbonate) hard block (*T*_g_ 120 °C).^116^ Because of the block chemistries’ similarity, very high molar mass values (*M*_*n*_ = 350 kg mol^–1^) were required to drive phase separation and elastomeric behavior was comparable to that of a soft rubber (σ_u_ = 2 MPa, *E*_*y*_ = 1.4 MPa, ε_b_ = 1052%). To avoid tapered junctions, the second epoxide (CHO) was only added to the reactor after complete conversion of the first (1-octene oxide). In future, the instantaneous selectivity change afforded by switchable catalysts could be an asset for such polycarbonate block TPEs.

Another opportunity is to make high-performance thermoplastics that compete with conventional resins or thermosets. Methods to improve TPE toughness include dynamic cross-linking strategies (*vide infra*), semicrystalline hard blocks, and judicious combinations of soft/hard blocks. The entanglement characteristics of the soft block polymer are important, and reducing the molar mass between entanglements (*M*_e_) can toughen elastomers.^117^ Applying switch catalysis to anhydride/epoxide/lactide combinations could deliver a low *T*_g_, high *M*_e_ anhydride/epoxide soft block combined with crystalline PLA hard domains.^75,81^ Such an approach may also overcome difficulties of scaling some of the specialist bioderived lactones currently used to deliver soft-block polyesters. Beyond PLA, which has a limited upper service temperature (55–60 °C), recent research shows the potential of carbohydrate derived polymers as potential hard segments.^118,119^ Furthermore, exploiting alkene substituents provides sites for postfunctionalization or chemical cross-linking. Toughened elastomers could be possible by introducing a small fraction of ionizable groups (i.e., an ionomer). For example, Filippidi et al. showed that adding just 14 mol % Fe(III)-catechol groups both toughened (×92) and improved the tensile strength (×58) of elastomeric networks comprised of poly(ethylene glycol), catechol and diamine cross-linker.^120^

## Pressure Sensitive Adhesives

Pressure-sensitive adhesives (PSAs), a class of nonreactive adhesives that bond to surfaces on gentle pressure application, are important in packaging, automotive, medical, and electronics industries.^121,122^ Apart from natural rubber-based PSAs, most formulations employ petrochemical polymers, including polyacrylates or styrenics. The drive toward circular plastics economies targets renewably sourced, degradable/recyclable PSAs with a lower environmental footprint. The oxygenated block polymers afforded by these switchable catalyses should show better controlled degradation chemistries, and their high oxygen content should improve substrate adhesion, particularly for glass, metal, and natural tissues.^123,124^ There is already good precedent for polyester-based PSAs, their potential being established by Long and co-workers.^125^ Grinstaff and co-workers later showed that CO_2_-derived polycarbonates are also promising PSA candidates.^126^ These materials could function in both dry and aqueous environments, and some samples showed thermoresponsive adhesion.^127^

**Figure 8:**
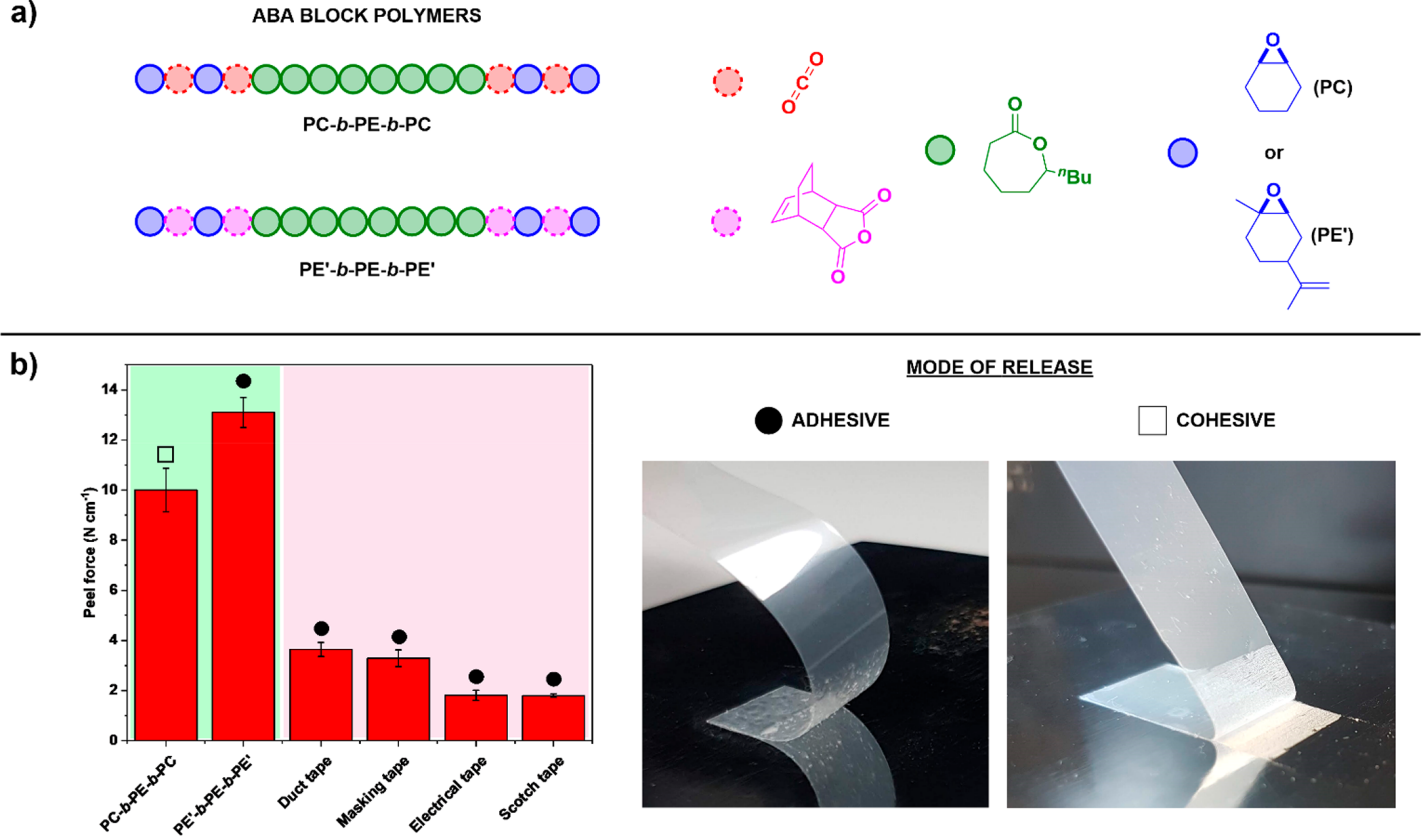
Pressure-Sensitive Adhesive (PSA) properties in triblock polymers made via switchable polymerization techniques. (a) Depiction of block structure for ABA triblock polymers comprised of polycarbonate (PC) and polyester (PE/PE’) blocks and the constituent monomers. (b) Peel strength of ABA block polymers compared to commercial adhesive tapes and illustration of different release modes.^71,73^ Image adopted with permission from ref (71). Copyright 2020 American Chemical Society.

Many successful PSAs have ABA triblock polymer structures featuring immiscible hard and soft segments; the soft matrix and the rigid domains strike a balance between viscous and elastic properties, which manifests as both surface wetting and shear resistance capabilities. Nevertheless, many of these polymers require further formulation with tackifiers and/or fillers to deliver the required adhesive behavior.^128−130^ While some additives can be renewably sourced (e.g., rosin ester), formulated products are necessarily more complex and may present particular challenges in future recycling and waste streams. One option would be to target oxygenated PSAs as single component adhesives. Our team recently reported carbon dioxide derived PCHC-*b*-PDL-*b*-PCHC triblock polymers, prepared by switchable catalysis, some of which showed high-shear, permanent PSA behavior (Figure 8).^71^ The peel force adhesion values obtained were competitive with several commercial adhesive tapes and with other literature PSAs. Using switchable catalysis and biobased tricyclic anhydrides (terpenes)/limonene oxide (citrus fruit peel)/ε-decalactone (castor oil) delivered a series of all-polyester PSAs, with easily controllable compositions (Figure 8).^73^ The leading PSAs, applied without fillers, showed high peel force values and dynamic mechanical properties throughout the PSA classification quadrants. The terpene derived hard block structures exemplify the potential for other natural (bio)chemistries to improve adhesive performances. In future judicious postfunctionalization of the pendent alkene moieties on the limonene oxides could further enhance adhesion, tailor substrate binding, and provide specific sites for interchain coupling or metal coordination.^131^ Both these new block polymer PSA classes will benefit from deeper investigation of rheological, adhesive, substrate scope, and long-term stability properties.

## Toughened Plastics

ROCOP catalyst developments have greatly expanded the range of carbon dioxide-derived polycarbonates, particularly those showing amorphous structures with high glass transition temperatures (e.g., cyclohexene/limonene oxide). However, these polymers can be very brittle and practical processing will require additives. In future, switchable catalysis could afford multiblock polymers with desirable combinations of rigidity and elastomeric properties. Such block copolymers might allow for tempering of brittleness, improved processability, and, importantly, enhanced mechanical properties. They could deliver better stand-alone materials or rubber toughening in blends. Three recent reports demonstrate the preparation of ductile plastics using these switchable catalyses. Rieger and co-workers prepared block poly(ester-carbonates), featuring PCHC or PLC (poly(limonene carbonate)) hard-blocks in combination with PHB (poly(hydroxy butyrate)) soft blocks.^80^ The Zn(II) β-diiminate catalyst (class **III**) was controlled between lactone ROP and carbon dioxide/epoxide ROCOP cycles, and changing the CO_2_ pressure yielded statistical or block copolymers. Hot-pressed polymer specimens subjected to tensile mechanical testing showed higher elongations at break than the polycarbonate homopolymers (PCHC or PLC). Elongation at break values ranged from 13% to 18%, with increased PHB content leading to greater extensibility. Benefits of the approach include the high block polymer molar mass values and the use of commercially relevant biodegradable PHB. Our group applied the Zn(II)Mg(II) catalyst (class **I**) to polymerize ε-decalactone, cyclohexene oxide, and carbon dioxide to make ABA triblocks with PCHC “A” blocks and a PDL “B” block.^71^ Optimizing the block compositions and molar mass values (>50 kg mol^–1^) resulted in block microphase separation. Lead samples, comprising 40–50 wt % polycarbonate, showed elongation at break values >900% and corresponding tensile toughness as high as 112 MJ m^–3^. Such values exceed the performances of either toughened PLA or commercial polycarbonate. In the future, it should be possible to increase the stress at yield and break values and diversify the soft-block composition by changing the monomers and increasing overall polymer molar mass. We also reported fully bioderived ABA type block polymers prepared from limonene oxide, carbon dioxide, and ε-decalactone. The leading plastic combines tensile strength (stress at break, σ_b_, = 21.2 MPa, Youngs Modulus, *E*_*y*_, = 321 MPa) and very high elasticity (elongation at break, ε_b_ = 400%)—an enhancement of more than 20× in elongation at break and tensile toughness over poly(limonene carbonate). It also undergoes selective, catalyzed depolymerization to limonene oxide, carbon dioxide, and the precursor polyester, providing a future chemical recycling and upcycling opportunity.^132^

## Solution Self-Assembled Block Polymers

One benefit of controlled polymerizations, such as heterocycle ROP/ROCOP, is the ability to readily apply initiators or chain transfer agents to access different polymer architectures. So far, switchable catalysis using mono-/bifunctional alcohols or carboxylic acids has delivered linear di- (AB) and triblock (ABA) polymers, respectively. One interesting future direction for AB polymers is exploring solution self-assembly and to target amphiphilic but degradable polymer blocks. We recently demonstrated amphiphilic polyesters, not made using switchable catalysis, that form polymersomes in aqueous or simulated biological fluids and could be interesting targets for controlled release applications with each block degrading completely to metabolites.^103^ The same principles which govern the self-assembly of conventional AB polymers will apply to these oxygenated polymers, and thus, intra- and inter-supramolecular interactions (H-bonding, solvophobicity, electrostatics, metal coordination) can also be exploited.^133^ Targeting hydrophilic polymer blocks is feasible by three different approaches: (1) Using polymeric chain transfer agents, e.g. hydroxyl-end-capped PEO; (2) Using switch catalysts active for epoxide ROP to yield hydrophilic polyethers, e.g. PEO; or (3) Introducing hydrophilic substituents by postfunctionalization reactions. The first concept is already very well-known in ROCOP catalysis and, naturally, also works well in switchable systems. The second remains under-explored but could be very attractive. In one exemplification, a single Cr(III) catalyst switched from PO/anhydride ROCOP to PO ROP to deliver poly(ester-*b*-ethers); although PPG is not hydrophilic, other epoxides could be substituted.^56^ Another report makes poly(ester-*b*-ethers) using a switchable organocatalyst for PA/EO ROCOP, followed by PO ROP.^134^ Careful assessment and management of safety is paramount when planning experiments using EO since it is toxic, volatile, and forms explosive mixtures in air. The third strategy might be particularly powerful, as it allows testing of multiple functional groups from a single backbone chemistry. The groups of Darensbourg and Coates have pioneered postfunctionalization of polycarbonates and -esters, from ROCOP, demonstrating clean and highly efficient coupling chemistries, such as the well-known thiol–ene reaction, to introduce water-soluble ether, carboxylic acid, or ammonium substituents.^44,105,115,135−139^

Darensbourg and co-workers showed that block polymer amphiphiles self-assemble in solution to form micelles and that fully water-soluble polymers are also accessible by appropriate control of substituent chemistry.^135,136^ Other postfunctionalization chemistries compatible with polyester or carbonate backbone chemistries include hydroboration/oxidation, Schiff base formation, Diels–Alder cyclization, and alkene epoxidation.^104,105^

Substituting polymer backbones with functional groups capable of dynamic cross-linking allows for adaptable, self-healing, and/or toughened materials. Indeed, the current status of this field with respect to dynamic covalent adaptable networks was recently reviewed by Sumerlin and co-workers.^140^ Recent work establishes that polycarbonates, from epoxide/CO_2_ ROCOP, featuring hydrogen bonding amide groups showed autonomous self-healing by rearrangement of the soft domains across a broken interface. The materials regenerated by reversible hydrogen bonding between the amide and carbonate groups.^139^ Other work employed glycerol substituents and diboronic ester as dynamic cross-linkers to impart self-healing capability to CO_2_-based TPEs at room temperature.^115^

Control over polymer architecture should also be explored using tri-, tetra-, and penta-hydroxyl initiators to form multiarm star polymers. Such structures are well-known products using lactone ROP and expand the mechanical property range.^141^ For example, 3-, 4- and 6-arm star poly(ε-decalactone)-poly(l-lactide) elastomers have higher Young’s moduli (3.7× for 6-arm) and stress at break (2× for 6-arm) than equivalent linear (2-arm) polymers.^142,143^ These switchable catalytic polymerizations should also be amenable to the formation of graft polymers by exploiting postfunctionalization or protection/deprotection strategies to expose hydroxyl decorated backbones that can initiate polymer chain growth. Aoshima and co-workers recently provided a demonstration of a single catalyst simultaneously effective for cyclic ester ROP and cationic vinyl-ether polymerization to deliver branched materials.^144^

## Higher-Order Block Sequences and Multiblocks

**Figure 9:**
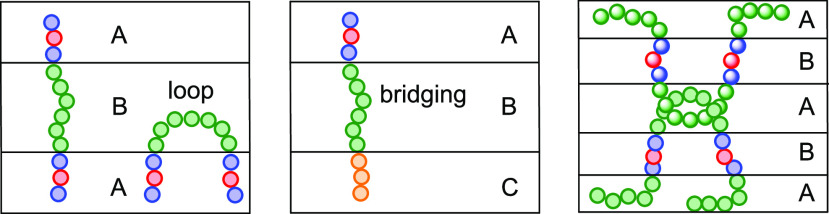
Illustration of different bridging, looping and knotting morphologies in ABA, ABC, and ABABA block copolymers.

**Figure 10:**
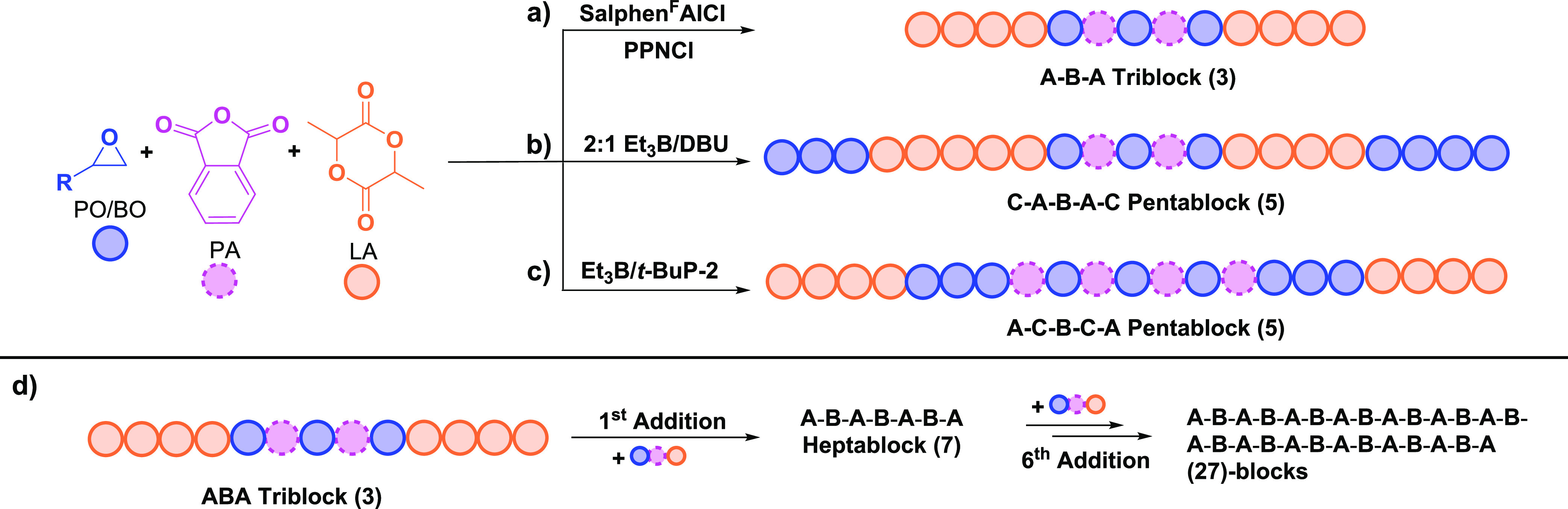
Multiblock polymers prepared using switch catalysis. (a) All polyester triblocks (PA/PO ROCOP and LA ROP) catalyzed by Al catalyst. (b) Organocatalysed formation of poly(ester-*b*-ester’-*b*-ether) pentablocks (PA/PO ROCOP, LA ROP, and PO ROP). (c) Organocatalysed formation of poly(ester-*b*-ether-*b*-ester′) pentablocks where following PA/PO ROCOP, PO ROP precedes LA ROP. (d) Example of construction of ABA sequence (27)-block polyesters using switch catalysis.^75,83^

Higher-order blocks and multiblock polymers may access a wider range of phase separated nanostructures and offer property improvements.^145−147^ For example, ABC triblock polymers, featuring three immiscible blocks, may promote morphologies where chains bridge distinct hard domains rather than loop back into the same domain (Figure 9). Increasing the density of bridge sites correlates with higher Young’s moduli, stress, and elongation at break. ABABA pentablock structures, on the other hand, form knotted looping structures in the central A block improving the mechanical resistance to deformation. This concept was exploited by Hillmyer and co-workers using PLA–PDL–PLA multiblocks, formed by coupling of the triblock polymers with diisocyanates, which were processable at lower temperatures than the triblock analogues and compatible with industry-relevant injection molding.^110^ The preparation of multiblock polymers can, however, entail increasingly lengthy and challenging syntheses. Further, synthetic methods that selectively form specific multiblock patterns without contamination by lower-order sequences (such as AB or ABC) are essential to study phase separation and improve properties. In this regard, the switch catalysis method helps access multiblock structures. Stoβer et al. prepared all polyester hepta- and, even, icosikaihepta(27)-block polymers featuring the repeat ABA sequence (Figure 10a).^75^ This was achieved using mixtures of propylene oxide (PO)/phthalic anhydride (PA)/racemic lactide (*rac*-LA), all of which are commercial monomers. The switch catalyst delivers ABA sequences because of the chain end group selectivity, and by sequential monomer mixture additions, multiple repeats of this sequence were delivered (Figure 10a and d). In this case, the choice of catalyst system, [Salphen^F^AlCl]/PPNCl, was critical since it limits block transesterification and does not form any ether linkages (no epoxide ROP).^105^

Using the same monomer mixture, an organocatalyst system (2:1 Et_3_B/DBU) produced a pentablock featuring polyether end-blocks (i.e poly(ether-*b*-ester-*b*-ester’-*b*-ester-*b*-ether) (Figure 10b).^83^ Conversely, pentablocks with the polyether block inserted between the polyester blocks (i.e., poly(ester-*b*-ether-*b*-ester′-*b*-ether-*b*-ester)) were produced using a different organocatalyst and butene oxide (BO) monomer (Et_3_B/*t*-BuP-2) (Figure 10c).^82^ The latter block sequences were also produced using a Cr(III) salen catalyst system ([SalcyCrCl]/[PPNCl]) exposed to tricyclic anhydride (TCA)/PO/DL mixtures. As mentioned above, pentablock polymers are also accessible by switch catalysts enchaining via two ROCOP cycles joined with a heterocycle ROP.^69^

Using the same monomer mixture, an organocatalyst system (2:1 Et_3_B/DBU) produced a pentablock featuring polyether end-blocks (i.e., poly(ether-*b*-ester-*b*-ester’-*b*-ester-*b*-ether) (Figure 10b).^83^ Conversely, pentablocks with the polyether block inserted between the polyester blocks (i.e., poly(ester-*b*-ether-*b*-ester’-*b*-ether-*b*-ester)) were produced using a different organocatalyst and butene oxide (BO) monomer (Et_3_B/*t*-BuP-2) (Figure 10c).^82^ The latter block sequences were also produced using a Cr(III) salen catalyst system ([SalcyCrCl]/[PPNCl]) exposed to tricyclic anhydride (TCA)/PO/DL mixtures. As mentioned above, pentablock polymers are also accessible by switch catalysts enchaining via two ROCOP cycles joined with a heterocycle ROP.^69^

Even at this nascent stage, it is clear that switchable catalysis can easily deliver a range of new multiblock sequences and chemistries; future understanding of how structures correlate with macroscopic properties will allow better targeting. A materials-focused approach should be driven by careful consideration of the best monomer combinations and most appropriate catalysts to target specific polymer properties and meet application need. One immediate area for development is to exploit multiple switchable polymerizations to build-up new multiblock structures featuring crystalline-glassy-rubbery-glassy-crystalline components to enhance tensile strength.^132,148^

## Polymer Degradation and Upcycling

**Figure 11:**
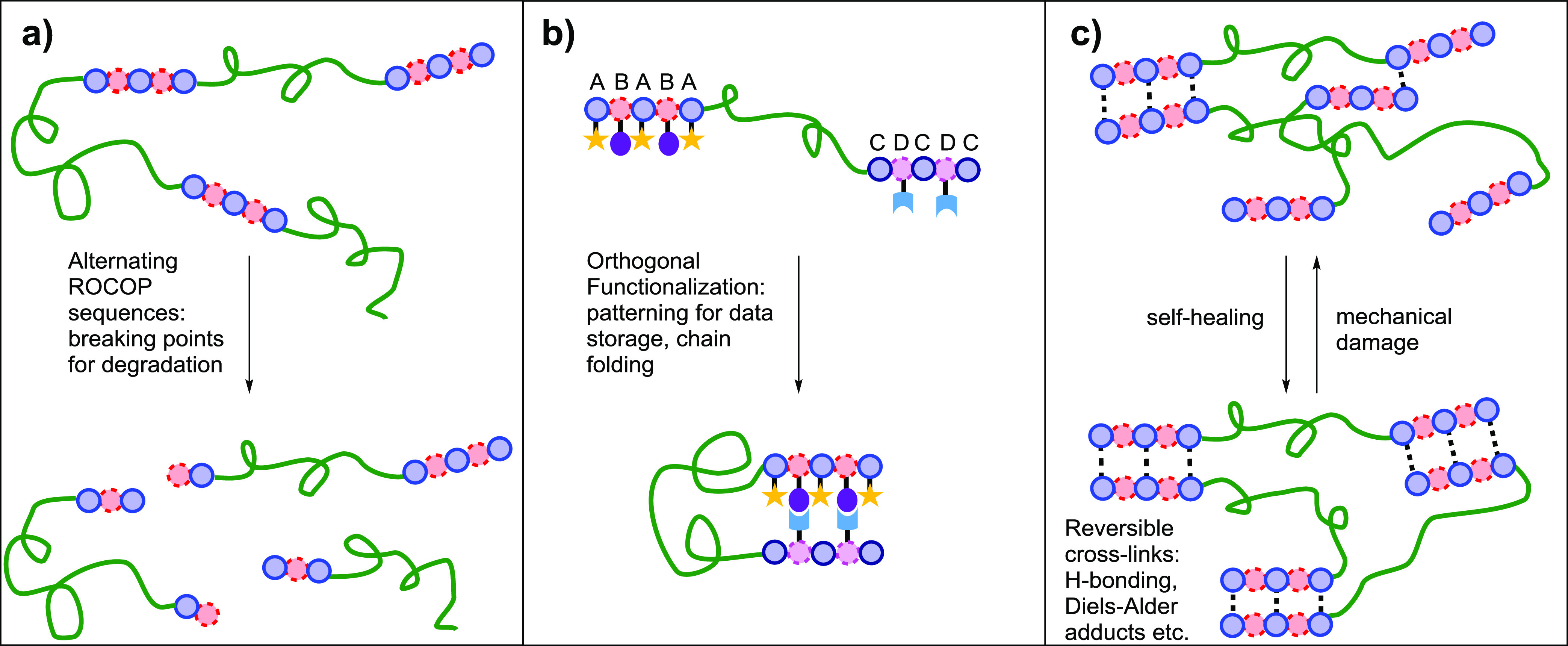
Future application targets for switch catalysis. (a) Designed degradation via the installation (by ROCOP) of ester or carbonate linkages susceptible to hydrolyses. (b) Chain folding and chemical data storage through selective functionalization. (c) Adaptable materials and enhanced properties through dynamic cross-linking.

Many of the new polymer chemistries produced by these switchable catalysts are targeted to facilitate polymer degradation and chemical recycling processes. There is growing literature demonstrating that finer control over ester/carbonate linkage chemistry improves degradation profiles. For example, Lui and Cui showed accelerated hydrolytic degradation rates for poly(lactide-*co*-carbonates), where structures featured a higher proportion of alternating carbonate-lactide linkages (random copolymers) compared to block or tapered structures.^149^ Prior to this, Meyer and co-workers used iterative syntheses to target specific lactide and glycolide linkages in polyester structures.^98,150,151^ They established that alternating PLGA structures yield gradual but constant degradation rates compared to random arrangements of the same overall composition. Significantly, in 2019, Meyer and co-workers showed that even using short segments of sequence precise units elicited a marked degradation rate impact.^152^ An important future direction would be to exploit switch catalysis to embed short sequences of precisely alternating polyester or -carbonate segments as regular breakpoints, perhaps within slower degrading polymer chains (Figure 11).

In the context of circular economy plastics, there is a growing interest in the chemical depolymerization of polymers; both polyesters and carbonates are well suited to solvolysis reactions that re-form small molecules/monomers with excellent yields.^2,153^ Recent advances in catalysis, amenable to the polymer types produced by switchable catalysis, have resulted in impressive activity and selectivity values.^154,155^ Many of the relevant polyesters/carbonates can also be chemically recycled to cyclic esters/carbonates—i.e. directly back to monomer mostly through catalyzed chain backbiting reactions occurring above the ceiling temperature (50–300 °C, depending upon structure).^156−160^ The special case of chemical recycling of polycarbonates, prepared by epoxide/CO_2_ ROCOP, is worth highlighting. Recently several of these polycarbonates have been found to undergo catalyzed depolymerization to reform epoxides and carbon dioxide.^132,161−166^ By combining different depolymerization chemistries, the selective breaking and reforming of new sequences should be feasible. As proof of principle, we recently degraded a triblock PLC–PDL-PLC by selective and quantitative polycarbonate depolymerization to extrude limonene oxide, carbon dioxide, and the central PDL block.^132^ Subsequently, the PDL block could either be degraded, for example, by solvolysis or used as a chain transfer agent in other polymerizations.

## Future Challenges and Opportunities

In summarizing the state of the art for these switchable polymerization catalyses, it is clear there are many opportunities for further research and development, and we have outlined these in themed focus groupings.

### 1 Switchable Processes

In its first phase, switch catalysis has successfully delivered block polymers featuring ester, ether, and carbonate chemistries. In future, these switches should be investigated using other heterocycles, particularly those featuring S-(COS, thiiranes, thioanhydrides) or *N*-heterocycles (*N*-carboxyanhydrides, aziridines, etc.).^167−500^ There has been a resurgence of interest in heteroatom functionalized polymers, and it is expected many of these currently under-explored monomer combinations will succeed in switch catalysis. Aside from increasing block incompatibility such heteroatom functionalized polymers may also modify the refractive index, scavenge heavy metals, coordinate catalysts, or mimic tertiary structural features of biopolymers, like α helices or β sheets.^172−176^

There remain several prosaic challenges around monomer purities, catalyst tolerance, and switchable processes to ensure the broadest applicability. More research into the scalable purification of reagents like carbon dioxide and cyclic anhydrides, particularly to remove residual water, may be necessary to drive multiblock polymer molar masses.^177^ On the other hand, catalysts that are tolerant to added, or contaminated, water offer an efficient and convenient means to deliver hydroxyl telechelic polycarbonate/ester polyols (2–5 kg mol^–1^).^39,178−180^ These products are used to make valuable higher polymers (e.g., polyurethanes), in resin chemistry and as surfactants.^30^ Future catalyst design should increase catalyst tolerance to impure monomers and large excesses of chain transfer agents.

### 2 Catalysis

Aside from the usual goals for catalysis of enhanced rates, reduced loading, increased selectivity, and tolerance, which are not trivial challenges on their own, switch catalysis demands advances across multiple catalytic cycles and requires the highest end-group fidelity. The ability to polymerize a wide range of monomers at sufficiently high rates across several cycles remains challenging. In particular, innovation is needed in heterocycle/heteroallene ROCOP catalysts since the diversity of structures remains quite limited.^29,181^ Improving the performances of organocatalysts should focus on maintaining the benefits of their high tolerance and monomer scope but significantly increasing rates and molar mass values, as well as ensuring operability at low catalyst loadings.^41,42,87^ In parallel, advances in synergic heterodinuclear catalysts should be explored since these species operate without cocatalysts, show high tolerance to water/alcohols, and operate at low carbon dioxide pressures.^38,39,91,182^ Recent advances using inexpensive, abundant, and lightweight metals such as sodium(I), potassium(I), and magnesium(II) are opportunities to lightweight switch catalysts.^39^ In 2020, a heterodinuclear Co(III)K(I) catalyst showed high activity in PO/CO_2_ ROCOP, with high stability to excess alcohol (up to 250 equiv), enabling broad molar mass control (1.5–80 kg mol^–1^).^39^ By exploiting synergic heterodinuclear catalysts, at least an order of magnitude rate enhancement compared to homodinuclear analogues is possible. For example, a Mg(II)Co(II) catalyst showed a turnover frequency for CO_2_/CHO ROCOP of 455 h^–1^ (0.05 mol %, 80 °C, 1 bar CO_2_).^38^

### 3 New Switches

As well as broadening the range of heterocycles, future switch catalysis must drive to access other polymerization mechanisms. The development of catalysts switchable between metal–carbon and metal–oxygen intermediates would provide access to polyacrylate, vinyl ether, and olefin blocks, as well as the oxygenates described in this perspective. A recent report describes a Co(II)salen catalyst for organometallic-mediated radical polymerization (OMRP) of vinyl acetate, followed by an oxidative switch, purification of the polymer, and its subsequent use in carbon dioxide/epoxide ROCOP. The switch occurs upon the addition of O_2_, which oxidizes the Co(II) to Co(III) and converts the cobalt–carbon bond to a cobalt–oxygen bond.^183^

Another highly promising area is stereoselective catalysts to produce crystalline blocks. Stereoselective epoxide/heterocumlene ROCOP catalysts can function using either enantiopure monomers or racemic mixtures.^184−190^ Many isotactic polyester/carbonates can form stereocomplexes by cocrystallization with the opposite enantiomer. Such stereocomplexes may show higher melting temperatures and mechanical properties than the isotactic analogue; the prototypical example is stereocomplexed PLA which shows a >50 °C higher melting temperature than PLLA.^191^ Stereocomplex PLC shows higher thermal stability (>∼14 °C increase on onset temperature) compared to the isotactic analogue. Stereocomplex poly(propylene succinate) exhibited an enhanced melting point of 120 °C, similar to low-density polyethylene than its isotactic parent (79 °C).^187^ Such stereocomplex portions can also be incorporated into triblock polymers, as demonstrated by PLLA/PDLA stereocomplexation in thermoplastic elastomers.^192^ Using triblock polyesters, featuring poly(trimethylene carbonate) PTMC soft blocks, the stereocomplexed materials showed a significantly higher Young’s Modulus (50% increase) compared to either isotactic variant, attributed to improved physical cross-linking.^193^ With polymenthide “soft” blocks, a tensile strength of 22 MPa was achieved for the stereocomplexed thermoplastic elastomer blends, versus only 2 MPa for the amorphous copolymers.^192^

### 4 New Materials and Applications

Many multiblock oxygenated polymer applications remain to be explored since this field of catalysis is still young. These polymers could be targeted for next generation battery electrolytes and binders,^194^ scaffolds for regenerative medicine,^195^ and/or stimuli responsive electroactive materials for use in soft-robotics. As society transitions to the widespread use of electric vehicles, there is growing impetus for better performing solid-state batteries to meet stricter safety and performance requirements. This presents an opportunity for solid polymer electrolytes comprising properly placed ether, carbonate, and/or ester blocks.^196^ The high degree of control over block sequences afforded by this catalysis may help decouple mechanical properties from ionic conductivity. The superior electrochemical stability of poly(carbonates/esters), compared to polyethers, also stimulates the preparation of materials with precise sequences and blocks.^197,198^ In regenerative medicine, poly(propylene fumarate) (PPF) allows for printable tissue-engineering scaffolds, whose final structures are “set” by cross-linking chemistry after printing. The polymers benefit from the intrinsic hydrolytic degradability and formation of nontoxic and resorbable byproducts.^195^ Becker and co-workers already applied a switchable Mg(II) catalyst to deliver PPF block oligomers, which were cross-linked during fabrication, to produce 3-D scaffolds.

Another interesting direction is to combine these multiblock polymers with natural fibers like cellulose, hemp, silks, or peptides. Exploiting amphiphilic polymers, prepared by these switchable polymerizations, could help to compatibilize natural fibers with bioderived matrices like PLA.^199^ Consumer concerns over the health effects and environmental leaching of plasticizers and the need for toughening of many bioderived materials suggest more attention should focus on multiblock systems as impact modifiers.

Finally, there are tremendous opportunities to improve the selection, design, and even processes to make these polymers that exploit the rapid advances in automation, machine learning, and robotics. The importance of optimizing polymer processing to deliver the best mechanical properties, advancements in flow chemistry, and a focus on scale-up will be essential to translate discoveries into products or applications.

The fascinating fundamental polymer chemistry and physics of alternating sequenced multiblock polymers also need to be fully investigated and understood. As set out in a recent perspective article from Sing, a better understanding of multiblock polymer physics will be central in directing which sequence-defined polymers to target next.^200^ Understanding precisely how material properties are dictated and controlled by parameters such as chain sequence order, length, and dispersity, as well as by interchain interactions, will empower future polymer design. We propose that these new switchable catalysts and polymerizations have a key role to play in advancing polymer theory, providing materials with controllable properties, diversifying the range of raw materials used away from petrochemistry, facilitating recycling and delivering high performance sustainable polymers.
